# Nonsense-associated altered splicing of *MAP3K1* in two siblings with 46,XY disorders of sex development

**DOI:** 10.1038/s41598-020-74405-1

**Published:** 2020-10-15

**Authors:** Maki Igarashi, Yohei Masunaga, Yuichi Hasegawa, Kenichi Kinjo, Mami Miyado, Hirotomo Saitsu, Yuko Kato-Fukui, Reiko Horikawa, Yomiko Okubo, Tsutomu Ogata, Maki Fukami

**Affiliations:** 1 Department of Molecular Endocrinology, National Research Institute for Child Health and Development, Tokyo, Japan; 2 Department of Pediatrics, Hamamatsu University School of Medicine, Hamamatsu, Japan; 3 Division of Urology, National Center for Child Health and Development, Tokyo, Japan; 4 Department of Biochemistry, Hamamatsu University School of Medicine, Hamamatsu, Japan; 5 Division of Endocrinology and Metabolism, National Center for Child Health and Development, Tokyo, Japan; 6 Department of Pediatrics, Shizuoka Saiseikai Hospital, Shizuoka, Japan; 7 Present Address: Laboratory of Health Nutrition, Department of Applied Biological Chemistry, Graduate School of Agricultural and Life Sciences, The University of Tokyo, Tokyo, Japan

**Keywords:** Genetics, Diseases, Endocrinology, Medical research, Molecular medicine

## Abstract

Although splicing errors due to single nucleotide variants represent a common cause of monogenic disorders, only a few variants have been shown to create new splice sites in exons. Here, we report an *MAP3K1* splice variant identified in two siblings with 46,XY disorder of sex development. The patients carried a maternally derived c.2254C>T variant. The variant was initially recognized as a nonsense substitution leading to nonsense-mediated mRNA decay (p.Gln752Ter); however, RT-PCR for lymphoblastoid cell lines showed that this variant created a new splice donor site and caused 39 amino acid deletion (p.Gln752_Arg790del). All transcripts from the variant allele appeared to undergo altered splicing. The two patients exhibited undermasculinized genitalia with and without hypergonadotropism. Testosterone enanthate injections and dihydrotestosterone ointment applications yielded only slight increase in their penile length. Dihydrotestosterone-induced *APOD* transactivation was less significant in patients’ genital skin fibroblasts compared with that in control samples. This study provides an example of nonsense-associated altered splicing, in which a highly potent exonic splice site was created. Furthermore, our data, in conjunction with the previous data indicating the association between MAP3K1 and androgen receptor signaling, imply that the combination of testicular dysgenesis and androgen insensitivity may be a unique phenotype of *MAP3K1* abnormalities.

## Introduction

Splicing errors caused by single nucleotide variants (SNVs) in exons or introns account for a substantial percentage of the etiology of monogenic disorders^1,2^. Previous studies have suggested that ~ 10% of disease-associated SNVs in exons result in aberrant splicing^3^, although these variants are typically recognized as missense, nonsense, or silent substitutions. These exonic splice variants usually cause exon skipping by disrupting a splice donor/acceptor site or an exonic splicing enhancer, and in some cases, lead to exonization of a part of intronic sequences by reducing the activity of a native splice site^1–5^. Theoretically, SNVs in exons can create new splice donor or acceptor sites and thereby lead to intronization of part of the exons^6^. However, this type of splice variants has rarely been reported. Abramowicz et al. documented that among 26 exonic splice variants in the *NF1* gene, only two generated new splice sites and caused partial exon deletion^1^.

The mitogen-activated protein kinase kinase kinase 1 gene (*MAP3K1*, NM_005921) is one of the causative genes for 46,XY disorders of sex development (DSD)^7^. To date, 19 pathogenic variants in *MAP3K1* have been submitted to the Human Gene Mutation Database (https://www.hgmd.cf.ac.uk/ac/index.php)^7–12^. The majority of these variants were missense substitutions in exons 1–13. Pathogenic *MAP3K1* variants are thought to exert gain-of-function effects, based on the results of in vitro functional assays performed for nine variants^8,12,13^. Patients with *MAP3K1* pathogenic variants characteristically present with complete or partial testicular dysgenesis, indicating that the proper function of MAP3K1 is critical for testicular development^7–12^. However, considering the small number of previous reports, much remains to be clarified for the spectrum of genetic defects and phenotypes of *MAP3K1* abnormalities. For example, Granados et al. reported an *MAP3K1* variant-positive patient who exhibited 46,XY DSD and normal blood levels of gonadotropins and anti-Müllerian hormone (AMH)^11^, raising the question of whether testicular dysgenesis is the sole mechanism of 46,XY DSD in patients with *MAP3K1* pathogenic variants. Here, we report two siblings with 46,XY DSD and a hitherto unreported *MAP3K1* variant. This study provides a novel example of splice variants and broadens the phenotypic spectrum of *MAP3K1* abnormalities.

## Results

### Identification of a rare ***MAP3K1*** variant in two siblings with 46,XY DSD

Patients 1 and 2 were male siblings who were identified through mutation screening for 110 patients clinically diagnosed with 46,XY DSD. In this screening, 11 major causative genes for 46,XY DSD were examined using a next-generation sequencer (NGS) panel. We called nonsense, frameshift, and splice site variants, as well as missense substitutions which were assessed as deleterious/pathogenic by two or more of four in silico programs. Variants whose allele frequency in the general population is more than 1% were excluded as polymorphisms. Variants of interest were confirmed by Sanger sequencing.

**Figure 1:**
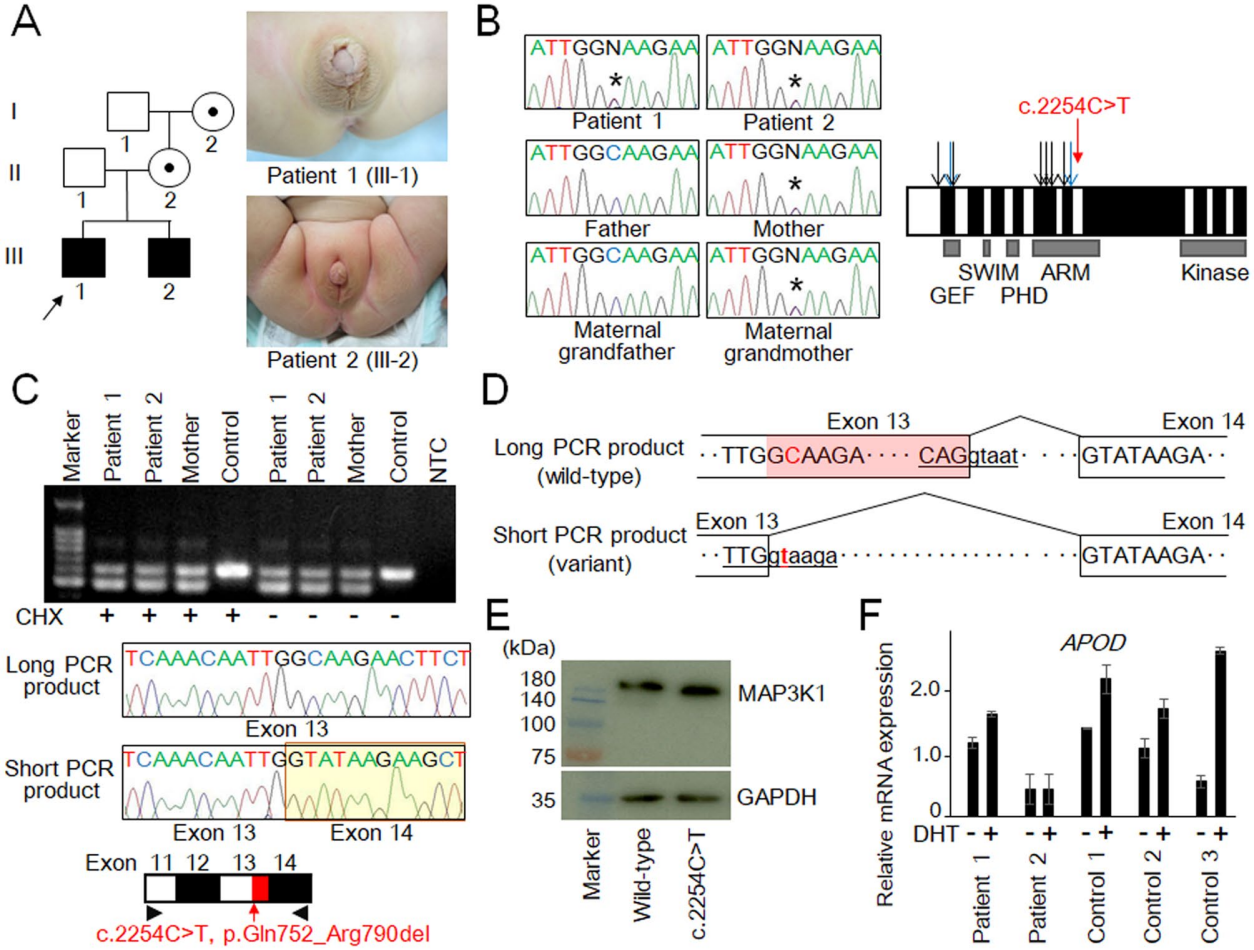
(**A**) Clinical information of the patients. Pedigree of the family and genital appearance of patient 1 (at 2.4 years of age) and patient 2 (at 6 months of age) are shown. (**B**) The c.2254C>T variant and known pathogenic variants in *MAP3K1*. Black and blue arrows indicate missense substitutions and other variants respectively, which have been subjected to in vitro functional assays^8,12,13^. White and black boxes denote odd- and even-numbered exons, respectively. GEF, guanine exchange factor domain; SWIM, SWI2/SNF2 and MuDR domain; PHD, plant homeo domain; ARM, armadillo repeat domain; and Kinase, protein kinase domain. Asterisks in the chromatographs depict the mutated nucleotide. (**C**) Representative results of RT-PCR. The upper panel shows the results of the patients, the mother, and an unaffected individual. NTC, negative control; and CHX, cycloheximide. The middle panel shows chromatograms of the long and short PCR products. The long PCR product was wild-type *MAP3K1*, while the short product was a variant transcript containing a 117-bp deletion in exon 13. The lower panel shows mRNA structures of the wild-type and variant *MAP3K1*. The red arrow delineates the position of the c.2254C>T variant, and the red box indicates the 117-bp deletion. Arrowheads depict the position of primers. (**D**) Predicted structures of the wild-type and variant transcripts. The uppercase and lowercase letters indicate nucleotides in exons and introns, respectively. The underlined letters indicate consensus sequences for a splice donor site^37^. The shaded region in the long PCR product indicates the 117-bp deletion. The affected nucleotide is shown in red. (**E**) Representative results of western blotting analysis. Transient transfection of the wild-type and variant MAP3K1 yielded proteins of the expected size and amount. GAPDH was used as a loading control. (**F**) The results of *APOD* induction assay. Expression levels of *APOD* relative to *GAPDH* (mean ± SD) are shown. Genital skin fibroblasts of the patients and control individuals were treated with either DHT (+) or methanol (DHT −).

As a result, we identified a c.2254C>T variant of *MAP3K1* in patients 1 and 2 (Fig. 1A,B), together with several other pathogenic gene variants in other patients (data not shown). The *MAP3K1* variant was recognized as a nonsense substitution (p.Gln752Ter) and was not found in the public databases. Sequence analysis of the patients’ family members revealed that the *MAP3K1* variant was shared by the mother and the maternal grandmother, and was absent from the unaffected male individuals in this family (Fig. 1A,B).

### Molecular analyses of patients 1 and 2

To exclude the possibility that 46,XY DSD of patients 1 and 2 resulted from a genetic or genomic abnormality other than the *MAP3K1* variant, we performed whole exome sequencing and array-based comparative genomic hybridization (CGH) analysis. Whole exome sequencing detected no other variant that could explain the phenotype of the patients (Table S1). Similarly, CGH analysis identified no pathogenic copy-number variations in the genome.

### Functional assessments of the *MAP3K1* variant

We analyzed the functional characteristics of the *MAP3K1* variant. Initially, this variant was recognized as a nonsense substitution in exon 13 that creates a premature termination codon (PTC) at the 752nd position in the armadillo repeat domain (p.Gln752Ter, Fig. 1B). Since the position of the PTC satisfied the condition for nonsense-mediated mRNA decay (NMD)^5^, the variant transcript was predicted to undergo early degradation. Hence, we analyzed total RNA samples extracted from immortalized lymphoblastoid cell lines of the patients and their mother. As a control, a sample from a cell line of an unaffected individual was also analyzed. RT-PCR was performed using a primer pair for exons 11 and 14 of *MAP3K1*. As shown in Fig. 1C, RT-PCR for the patients and their mother yielded products of two different sizes, while that for the control individual generated products of only one size. Sequence analysis revealed that the long and short PCR products of the patients and their mother were wild-type *MAP3K1* and a variant transcript lacking 117 nucleotides of exon 13, respectively (Fig. 1C,D). These results indicate that the c.2254C>T variant created a new splice donor site in exon 13, and thereby caused intronization of the 3ʹ-part of this exon (Fig. 1D). Thus, this variant appeared to result in an in-frame deletion of 117 nucleotides in exon 13, that is, 39 amino acid deletion within the armadillo repeat domain (p.Gln752_Arg790del). Western blot analysis using cells transiently transfected with MAP3K1 expression vectors showed that the variant allele produced a protein of the expected size and amount (Fig. 1E). The variant was assessed as “likely pathogenic” according to American College of Medical Genomics guidelines (PM1, PM2, PM4, and PP1)^14^. Finally, we examined whether the *MAP3K1* variant encodes an additional transcript that undergoes NMD. To this end, we treated the lymphoblastoid cell lines with cycloheximide, an inhibitor of NMD^15,16^. The results of RT-PCR remained unchanged after cycloheximide treatment (Fig. 1C). Specifically, we detected no transcripts containing the PTC.

### In silico splice site prediction

To examine whether the c.2254C>T variant formed a new consensus sequence of a splice donor site, we performed in silico analysis using five programs. As shown in Table 1, the variant sequence was recognized as a splice donor site by all in silico programs, while the wild-type sequence and the other nucleotide substitutions at the same position (c.2254C>A and c.2254C>G) were predicted to have no splice site activity.

**Table 1:** In silico prediction of splice site activity for the wild-type and variant sequences.

Program	Default threshold	Score of the wild-type sequence	Score of the variant sequence (c.2254C>T)	Score of c.2254C>A	Score of c.2254C>G
NNSPLICE	0.40	Not recognized as a splice site	1.00	Not recognized as a Splice site	Not recognized as a splice site
ASSP	4.50	Not recognized as a splice site	12.34	Not recognized as a splice site	Not recognized as a splice site
HSF	80.00^a^	62.92	90.06	No potential alteration of splicing	No potential alteration of splicing
MaxEnt	5.00^a^	1.09	8.85	0.66	1.20
SpliceRover	No data	Not recognized as a splice site	0.931558	Not recognized as a splice site	Not recognized as a splice site

NNSPLICE, Splice Site Prediction by Neural Network (https://fruitfly.org/seq_tools/splice.html); ASSP, Alternative Splice Site Predictor (https://wangcomputing.com/assp/); HSF, Human Splicing Finder (https://hsf.genomnis.com/home); MaxEntScan (https://hollywood.mit.edu/burgelab/maxent/Xmaxentscan_scoreseq.html); and SpliceRover (https://bioit2.irc.ugent.be/splicerover/).

^a^These thresholds are based on the previous report by Piton et al.^38^.

### Clinical features of patients 1 and 2

We examined clinical records of patients 1 and 2. These individuals were born to non-consanguineous healthy parents (Fig. 1A). Allegedly, there was no additional DSD patient in this family. The mother and maternal grandmother had no brother. The proband (patient 1) exhibited hypospadias and micropenis at birth. He underwent urethroplasty and chordee repair at 11 months of age and received testosterone enanthate (TE) injection (25 mg/dose, 3 times) from 1.9 years of age. Then, he underwent the second urethroplasty at 2.1 years of age. At 2.4 years of age, he was referred to our hospital. Physical examination revealed hypospadias and borderline micropenis (Fig. 1A and Table 2), while endocrinological evaluation showed age-appropriate levels of gonadotropins, testosterone, and AMH (Table 2). He was otherwise healthy and had no uterus or vagina. He was subjected to further TE injection (25 mg/dose) and also treated with topical dihydrotestosterone (DHT) ointment for 3 months. However, these treatments barely increased his penile length (Table 2).

**Table 2:** Clinical findings of the two patients.

	Patient 1 (III-1)	Patient 2 (III-2)	Reference range
Karyotype	46,XY	46,XY	
Social sex	Male	Male	
Present age	7.8 years	5.5 years	
**Age at examination**	2.4 years	6 months	
Genital findings			
Testis size	1.0 mL (bilateral)	< 1.0 mL (bilateral)	1.3 ± 0.3
Hypospadias	Yes (penile type)	Yes (penile type)	
Chordee	Yes	No data	
Cryptorchidism	No	Bilateral (inguinal)	
Penile length	2.5–3.0 cm	1.8 cm	3.3 ± 0.4 (for patient 1); 3.1 ± 0.4 (for patient 2)
	(after TE 25 mg i.m., 3ʹ)	(after TE 25 mg i.m., 3ʹ)	
Uterus/vagina	Absent on MRI	Absent on MRI	
Endocrine findings			
LH (IU/mL)			
Baseline^a^	< 0.3	6.6	< 0.4
Peak^a^	1.1	108.7	0.4–6.0
FSH (IU/mL)			
Baseline^a^	1.1	71.3	0.6–3.0
Peak^a^	8.0	238.5	6.3–15.6
Testosterone (nmol/L)			
Baseline^b^	< 0.10	1.56	0.10–0.45
Stimulated^b^	5.79	3.50	> 6.93
DHT (nmol/L)			
Baseline^b^	Not measured	Not measured	
Stimulated^b^	0.18	0.24	No reference
T/DHT ratio			
Stimulated^b^	9.3	4.2	< 10.5
AMH (pmol/L)			
Baseline	449.3	16.4	699 ± 245 (for patient 1); 793 ± 264 (for patient 2)
**Treatment**			
Orchidopexy	No	1.0 and 2.2 years	
Urethroplasty	11 months and 2.1 years	3.2 years	
Chordee repair	11 months	No	
Penile length after Tx	3.0 cm at 3.5 years after further Tx with TE 25 mg i.m., 1ʹ and DHT topical ointment for 3 months	2.3 cm at 2.3 years after further Tx with TE 25 mg i.m., 2ʹ and DHT topical ointment for 5 months	3.4 ± 1.0 (for patient 1); 3.3 ± 0.4 (for patient 2)

TE, testosterone enanthate; MRI, magnetic resonance imaging; LH, luteinizing hormone; FSH, follicle stimulating hormone; T, testosterone; DHT, dihydrotestosterone; AMH, anti Müllerian hormone; and Tx, treatment.

^a^Basal and peak values during a gonadotropin releasing hormone stimulation test (100 µg/m^2^ [max. 100 µg] bolus i.v.; blood sampling at 0, 30, 60, 90, and 120 min).

^b^Basal and stimulated values in a human chorionic gonadotropin stimulation test (3000 IU/m^2^/dose [max. 5000 IU] i.m. for 3 consecutive days; blood sampling on days 1 and 4).

Patient 2 was the younger brother of patient 1. Patient 2 manifested hypospadias, bilateral cryptorchidism, and micropenis at birth. He received TE injection (25 mg/dose, 3 times) from 2 months of age, and underwent orchidopexy. He was referred to our hospital at 6 months of age with his brother (patient 1). Physical examination revealed hypospadias, cryptorchidism, and micropenis without uterus or vagina (Table 2, Fig. 1A). Endocrine examinations revealed typical features of testicular dysfunction, i.e., a low AMH level, an impaired testosterone response to human chorionic gonadotropin stimulation, and markedly elevated levels of basal and stimulated gonadotropins (Table 2). Further TE injection (25 mg/dose, two times) and topical DHT ointment application for 5 months yielded only ~ 0.5 cm increase in the penile length (Table 2). The patient underwent orchidopexy at 1.0 and 2.2 years of age, and urethroplasty at 3.2 years of age.

At their latest visit, patients 1 and 2 were 7.8 and 5.5 years of age, respectively. They were healthy. Testicular ultrasound examinations showed no signs of tumor. Testicular biopsy was not conducted for these individuals.

### *APOD* induction assay using genital skin fibroblasts of the patients

The clinical features of patients 1 and 2 were indicative of a compromised response of the androgen receptor (AR) to androgens. Thus, to predict the function of AR, we conducted an *APOD* induction assay using cultured genital skin fibroblasts^17^. As a result, we found that DHT-induced *APOD* transactivation was less significant in cells of patients 1 and 2 compared with that in cells of three control individuals (Fig. 1F).

## Discussion

We identified a “likely pathogenic” *MAP3K1* variant in two siblings with 46,XY DSD. The c.2254C>T variant was initially recognized as a nonsense substitution leading to NMD. However, the results of RT-PCR suggested that this variant created a novel splice donor site which caused an in-frame deletion of 39 amino acids in the armadillo repeat domain. In this regard, previous studies of pathogenic *MAP3K1* variants have shown that several missense substitutions in the armadillo repeat domain exert high in vitro activity^12^. Thus, the 39 amino acid deletion in this domain may also enhance the function of MAP3K1, although this notion needs to be validated in future studies. Of note, the c.2254C>T variant corresponds to nonsense-associated altered splicing (NAS), one of the mRNA surveillance mechanisms to protect against the deleterious effects of PTCs^5^. To date, only a few cases with NAS have been reported^18–22^. Most of these cases caused exon skipping; however, in one case^21^, NAS created a new splice donor site similar to that in our case. Our results indicate that NAS can generate a mutant protein with a unique functional property.

It is worth mentioning that no PTC-containing transcript was detected in the patients’ cell lines even after cycloheximide treatment. These findings indicate that virtually all transcripts from the variant-positive allele were spliced at the new splice donor site. Consistent with this, the five in silico programs invariably predicted that the variant sequence is a splice donor site, whereas the wild-type sequence and the other two nucleotide substitutions at the same position have no splice site activity. These data suggest that in silico analyses are useful to identify splice mutations among all exonic SNVs. Moreover, our results provide evidence that an SNV can create a highly potent splice site in a genomic region, which usually has no splice site activity. However, given the limited number of previous reports^18–22^, generation of new exonic splice sites by SNVs, including NAS, appears to be a rare event in the human genome. Since it is known that splice site activity is determined both by the local sequence and by the genomic environment^23^, the new splice site in our case may be associated with favorable genomic circumstances.

The present study provides novel insight into the clinical manifestation of *MAP3K1* abnormalities. While patient 2 exhibited hypergonadotropic hypogonadism similar to that of previously reported patients with pathogenic *MAP3K1* variants^7–11^, patient 1 manifested undermasculinized external genitalia in combination with normal blood levels of gonadotropins and AMH. The phenotype of patient 1 is more consistent with impaired androgen sensitivity or defective genital organogenesis than testicular dysgenesis. Such a phenotype has also been observed in one patient with the *MAP3K1* p.Ala5dup variant (the proband of Family 1 reported by Granados et al*.*^11^). Importantly, micropenis of our patients was barely improved by TE injections and DHT ointment applications. The poor response of these patients to TE injections is in sharp contrast to the data of other cases with hypospadias or micropenis of various etiologies; these cases typically exhibit penile increment of 0.3–0.6 cm per injection^24,25^. It is known that most patients with micropenis due to testosterone deficiency are highly responsive to androgen treatment^26^. Therefore, although there is no doubt that testicular dysgenesis is the major cause of 46,XY DSD in patients with *MAP3K1* pathogenic variants^7–11^, other steps of male sex development may also be affected in these patients. In particular, *MAP3K1* abnormalities may perturb the AR signaling in the developing genitalia, because previous studies documented signal crosstalk between MAP3K1 and AR in prostate cancer^27,28^ and altered *AR* expression of cells transfected with a mutant MAP3K1^29^. Consistent with this, DHT-induced *APOD* transactivation, a biological marker of AR function^17^, was less significant in cultured genital skin fibroblasts of our patients compared with that in cells of the control individuals. However, since there is no previous report of TE injections or DHT ointment applications for patients with *MAP3K1* pathogenic variants, it remains unknown whether an impaired response to exogenous androgens is a consistent feature of these patients. Moreover, the precise role of MAP3K1 in the AR signaling pathway is yet to be clarified.

In summary, this study provides a novel example of NAS, in which a highly potent splice donor site was created in an exon. Furthermore, our data, in conjunction with the previous data indicating the association between MAP3K1 and AR signaling^27–29^, imply that the combination of testicular dysgenesis and androgen insensitivity may be a unique phenotype of patients with *MAP3K1* abnormalities.

## Methods

### Ethical approval

The study was approved by the Institutional Review Board Committee at the National Center for Child and Development and performed after obtaining informed consent from the patients’ parents. All methods were performed in accordance with the relevant guidelines and regulations.

### Mutation screening for 110 patients with 46,XY DSD

Mutation screening was performed for 110 patients clinically diagnosed with 46,XY DSD. We analyzed 11 major causative genes for 46,XY DSD (*AR, CBX2, DHH, GATA4, MAP3K1, NR5A1, SOX9, SRY, SRD5A2, WT1,* and *ZFPM2*) using a custom made NGS gene panel. The library was constructed using a HaloPlex kit (Agilent Technologies, Santa Clara, CA, USA) and sequence data were obtained using MiSeq or HiSeq 2000 (Illumina, San Diego, CA, USA). The sequencing reads were mapped by BWA (version 0.7.12) using Human GRCh37/hg19 (UCSC Genome Browser) as the reference. Base quality calibration was carried out by GATK (version 3.5). Functional consequences of missense variants were assessed using four in silico programs, i.e., Combined Annotation Dependent Depletion (CADD, https://cadd.gs.washington.edu/), PolyPhen-2 (https://genetics.bwh.harvard.edu/pph2/), Sorting Intolerant From Tolerant (SIFT, https://sift.jcvi.org/), and MutationTaster (https://www.mutationtaster.org/). Variants of interest were subjected to Sanger sequencing. The frequency of variants in the general population was examined by the Genome Aggregation Database (genomAD, https://gnomad.broadinstitute.org/), the Human Genetic Variation Database (https://www.hgvd.genome.med.kyoto-u.ac.jp/) and the 2,049 Japanese genome reference panel (2KJPN)^30^.

### Whole exome sequencing for patients 1 and 2

Whole exome sequencing was carried out for patients 1 and 2 using the SureSelect Human All Exon V6 kit (Agilent Technologies) and HiSeq 2000. The methods of NGS data analysis are described above. We referred to the NCBI database (https://www.ncbi.nlm.nih.gov/) to examine whether detected variants have previously been associated with 46,XY DSD.

### Array-based CGH analysis

CGH analysis was performed using a catalog human array (4 × 180 K format, Agilent Technologies). The results were analyzed using Agilent Genomic Workbench 7.0 (Agilent Technologies). We referred to the Database of Genomic Variants (https://dgv.tcag.ca/dgv/app/home) to exclude benign copy-number variations.

### RT-PCR using lymphoblastoid cell lines

Epstein-Barr virus transformed lymphoblastoid cell lines were generated from peripheral blood samples of patients 1 and 2, the mother, and an unaffected control individual. Total RNA was extracted from the cell lines using the RNeasy Mini Kit (QIAGEN, Hilden, Germany). RT-PCR was performed by SuperScript III Reverse Transcriptase (ThermoFisher Scientific, Waltham, MA, USA) and KOD One (TOYOBO, Tokyo, Japan) using a primer pair on *MAP3K1* exons 11 (5′-CCAGCCAGTTGTAGACACC-3′) and 14 (5′-TGTCCTGTTGACCATCCAAA-3′). RT-PCR products were subjected to Sanger sequencing.

Furthermore, to detect transcripts that are subjected to NMD, we treated the cell lines with cycloheximide^15^. The cells were cultured in media containing 100 μg/mL cycloheximide (Sigma-Aldrich, St. Louis, MO, USA) for 8 h before harvesting.

### Western blot analysis of transfected MAP3K1

An expression vector for the full-length wild-type *MAP3K1* (FHC3013E) was purchased from Kazusa DNA Research institute (Chiba, Japan). An expression vector for the c.2254C>T variant was created by mutagenesis using the In-Fusion HD cloning kit (Takara Bio, Shiga, Japan). These vectors were transfected into 293T cells by electroporation using the 4D-Nucleofector System (Lonza, Allendale, NJ, USA). We used the pDsRed-Monomer-N1 vector (Takara Bio) to confirm the transfection efficacy. After 24 h culture, the cells were harvested and subjected to protein extraction. Western blotting was performed by standard protocols. MAP3K1 proteins were detected using an anti-MEKK1 antibody (A302-395A, Thermo Fisher Scientific). As a loading control, GAPDH was detected using an antibody (D16H11, Cell Signaling Technology, Danvers, MA, USA). The entire procedure was performed twice.

### In silico splice site prediction

To examine whether the c.2254C>T sequence is consistent with a splice donor site, we performed in silico analysis using five programs, i.e., Splice Site Prediction by Neural Network (NNSPLICE, https://fruitfly.org/seq_tools/splice.html)^31^, Alternative Splice Site Predictor (ASSP, https://wangcomputing.com/assp/)^32^, Human Splicing Finder (HSF, https://www.umd.be/HSF3/)^33^, MaxEntScan (https://hollywood.mit.edu/burgelab/maxent/Xmaxentscan_scoreseq.html)^34^ and SpliceRover (https://bioit2.irc.ugent.be/splicerover/)^35^. For HSF and MaxEntScan, we submitted the variant information and 9 bp sequence around the variant, respectively. For the remaining programs, we submitted a 2,064 bp sequence consisting of intron 12, exon 13, intron 13 and exon 14 of *MAP3K1*. We also predicted the splice site activity of the wild-type sequence and the other nucleotide substitutions at the same position (c.2254C>A and c.2254C>G).

### *APOD* induction assay using genital skin fibroblasts

Genital skin samples were obtained from patients 1 and 2 during surgery for hypospadias. The *APOD* induction assay was performed as described previously^36^. In brief, total RNA samples were extracted from cultured genital skin fibroblasts after 72 h treatment with DHT (final concentration of 1 nM, Sigma-Aldrich) or methanol. Expression levels of *APOD* were measured by real-time PCR using a TaqMan gene expression assay kit (Hs00155794_m1; Thermo Fisher Scientific). *GAPDH* (4326317E; Thermo Fisher Scientific) was used as the internal control. As reference samples, genital skin fibroblasts obtained from three boys with buried penis were utilized. The assay was performed in duplicate for each sample and repeated twice.

## Supplementary information

Supplementary Information.

## Data Availability

The datasets generated during and/or analyzed during the current study are available from the corresponding author on reasonable request.
