# Supplementary material for: Nonsense-associated altered splicing of MAP3K1 in two siblings with 46,XY disorders of sex development
Source: Sci Rep. 2020 Oct 15;10:17375. doi: 10.1038/s41598-020-74405-1 (PMC7567082; doi:10.1038/s41598-020-74405-1)
Supplement: Supplementary file 1 — Supplementary Information. [file 41598_2020_74405_MOESM1_ESM.pdf]

Nonsense-associated altered splicing of *MAP3K1* in two siblings with 46,XY disorders of sex development

Maki Igarashi[1†], Yohei Masunaga[2], Yuichi Hasegawa[3], Kenichi Kinjo[1,2], Mami Miyado[1], Hirotomo Saitsu[4], Yuko Kato-Fukui[1], Reiko Horikawa[5], Yomiko Okubo[6], Tsutomu Ogata[2*] & Maki Fukami[1*]

[1]Department of Molecular Endocrinology, National Research Institute for Child Health and Development, Tokyo, Japan.

[2]Department of Pediatrics, Hamamatsu University School of Medicine, Hamamatsu, Japan.

[3]Division of Urology, National Center for Child Health and Development, Tokyo, Japan.

[4]Department of Biochemistry, Hamamatsu University School of Medicine, Hamamatsu, Japan.

[5]Division of Endocrinology and Metabolism, National Center for Child Health and Development, Tokyo, Japan.

[6]Department of Pediatrics, Shizuoka Saiseikai Hospital, Shizuoka, Japan.

[†] Present address: Laboratory of Health Nutrition, Department of Applied Biological Chemistry, Graduate School of Agricultural and Life Sciences, The University of Tokyo, Tokyo 113-8657, Japan.

Correspondence and requests for materials should be addressed to: M.F. (fukami-m@ncchd.go.jp) or T.O. (tomogata@hama-med.ac.jp)

Supplementary figure 1.

The full gel image of RT-PCR analysis (Figure 1C). Lane 1, molecular weight marker; lanes 2 and 6, patient 1; lanes 3 and 7, patient 2; lanes 4 and 8, the mother; lanes 5 and 9, an unaffected individual; and lane 10, negative control. Samples for lanes 2-5 were treated with cycloheximide.

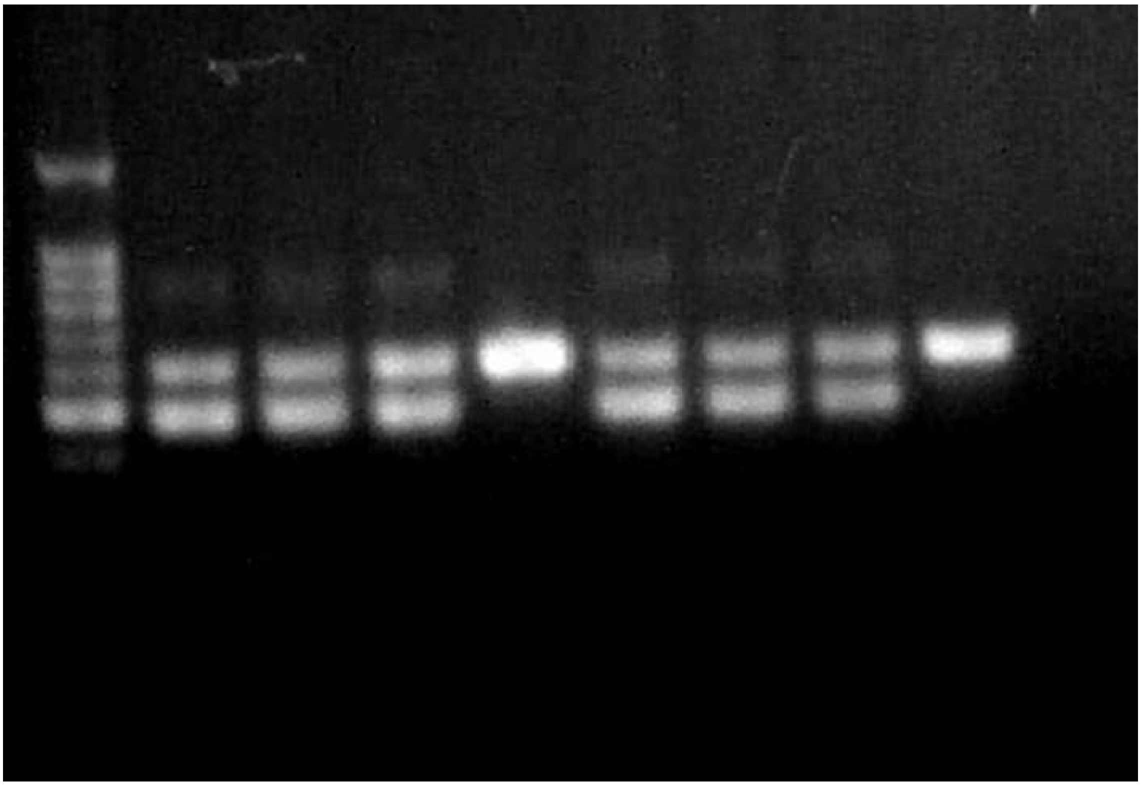

Supplementary figure 2.

The full length gel image of western blot analysis (Figure 1E). Three lanes in the left panel show the results of cells expressing the wild-type (lane 1) and variant MAP3K1 (lanes 2 and 3, cells from two independent transfections).

Three lanes in the right panel show the results of cells transiently transfected with GFP-MAP3K1 expression vectors. Because the poor expression of MAP3K1 is indicative of poor transfection efficacy of the GFP-tagged vectors, we did not utilized these vectors for further analysis.

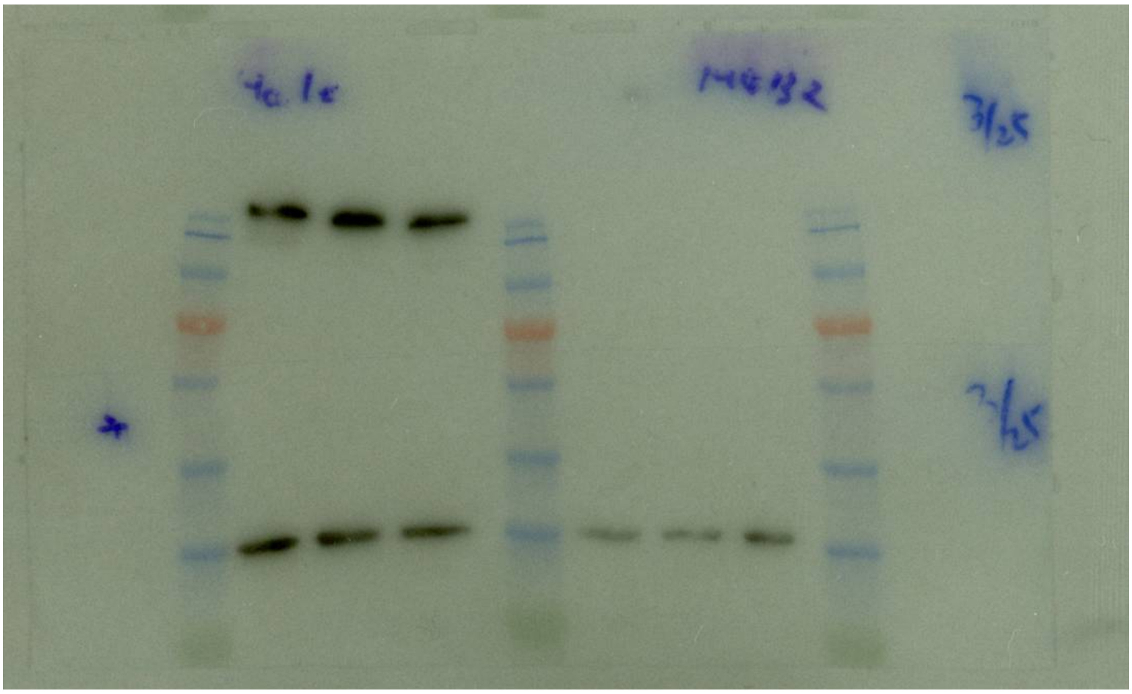

Table S1. Rare variants of patients 1 and 2 identified by whole exome analysis

Identified variant		Frequency in public databases (MAF)					*In silico* pathgenic analyses				Known disease	
Gene<br>(Location)	Variant	gnomAD_<br>genome_EAS*	gnomAD_<br>exome_EAS*	HGVD	2KJPN	In-house	CADD<br>PHRED score	PP2_HDIV<br>score	SIFT<br>score	MutationTaster<br>score	OMIM	Inheritance
**<Variants completely absent from the public and in-house databases>**												
MAP3K1<br>(5q11.2)	NM_005921.1:c.2254C>T:<br>p.(Gln752*)	None	None	None	None	None	1% most deleterious<br>40.0	–	–	Disease causing<br>1.000	46,XY sex reversal 6 (#613762)	AD
IPO11<br>(5q12.1)	NM_001134779.1:c.2080G>C:<br>p.(Asp694His)	None	None	None	None	None	1% most deleterious<br>29.1	Probably damaging<br>1.000	Damaging<br>0.003	Disease causing<br>1.000		
PDE8B<br>(5q13.3)	NM_003719.3:c.1998C>A:<br>p.(Phe666Leu)	None	None	None	None	None	1% most deleterious<br>24.4	Probably damaging<br>1.000	Damaging<br>0.000	Disease causing<br>1.000	Striatal degeneration (#609161)	AD
NPY<br>(7p15.3)	NM_000905.3:c.58G>A:<br>p.(Val20Met)	None	None	None	None	None	1% most deleterious<br>24.0	Possibly damaging<br>0.630	Damaging<br>0.014	Polymorphism<br>0.904		
TBATA<br>(10q22.1)	NM_001318241.1:c.547_570del<br>:	None	None	None	None	None	–	–	–	–		
MRPL17<br>(11p15.4)	NM_022061.3:c.327G>T:<br>p.(Gln109His)	None	None	None	None	None	Deleterious<br>19.1	Benign<br>0.003	Tolerated<br>0.155	Disease causing<br>0.956		
C2CD5<br>(12p12.1)	NM_001286174.1:c.1166A>C:<br>p.(Asp389Ala)	None	None	None	None	None	1% most deleterious<br>32.0	Benign<br>0.527	Damaging<br>0.002	Disease causing<br>1.000		
SPAG5<br>(17q11.2)	NM_006461.3:c.2300A>C:<br>p.(Gln767Pro)	None	None	None	None	None	1% most deleterious<br>23.7	Probably damaging<br>0.993	Damaging<br>0.000	Polymorphism<br>0.810		
**<Variants not completely absent from the public and in-house databases>**												
SCNN1D<br>(1p36.33)	NM_001130413.3:c.236C>T:<br>p.(Pro79Leu)	None	None	0.0024	0.0013	0.0023	Non-deleterious<br>0.011	Benign<br>0.000	Damaging<br>0.000	Polymorphism<br>1.000		
ENO1<br>(1p36.23)	NM_001428.5:c.985G>A:<br>p.(Ala329Thr)	None	0.0000<br>(0.000004061)	None	None	0.0023	Deleterious<br>14.4	Benign<br>0.002	Tolerated<br>0.074	Disease causing<br>1.000		
MFN2<br>(1p36.22)	NM_014874.3:c.13T>G:<br>p.(Phe5Val)	None	None	None	None	0.0023	1% most deleterious<br>25.1	Possibly damaging<br>0.993	Damaging<br>0.007	Disease causing<br>1.000	Charcot-Marie-Tooth disease 2a (#609260)	AD
EPHA2<br>(1p36.13)	NM_004431.4:c.1382C>A:<br>p.(Pro461Gln)	None	0.0003	0.0014	None	0.0023	Non-deleterious<br>1.47	Benign<br>0.118	Tolerated<br>0.598	Polymorphism<br>1.000	Cataract 6, multiple types (#116600)	AD
ITGA10<br>(1q21.1)	NM_003637.4:c.3223C>G:<br>p.(Arg1075Gly)	None	None	None	None	0.0023	1% most deleterious<br>23.2	Probably damaging<br>0.955	–	–		
CHD1L<br>(1q21.1)	NM_004284.5:c.2345T>C:<br>p.(Leu782Ser)	None	0.0007	0.0027	0.0047	0.0023	1% most deleterious<br>29.6	Probably damaging<br>0.992	Damaging<br>0.001	Disease causing<br>0.996		
DUSP12<br>(1q23.3)	NM_007240.2:c.217G>A:<br>p.(Glu73Lys)	0.0031	0.0037	0.0035	None	0.0023	Deleterious<br>16.6	Possibly damaging<br>0.948	Tolerated<br>0.348	Polymorphism<br>1.000		
DUSP12<br>(1q23.3)	NM_007240.2:c.218A>T:<br>p.(Glu73Val)	0.0031	0.0037	0.0035	None	0.0023	Deleterious<br>15.1	Possibly damaging<br>0.867	Tolerated<br>0.064	Polymorphism<br>1.000		
GPR37L1<br>(1q32.1)	NM_004767.4:c.1171C>T:<br>p.(Arg391Cys)	0.0000<br>(0.00006466)	0.0000<br>(0.000004062)	None	None	None	1% most deleterious<br>29.4	Probably damaging<br>0.999	Damaging<br>0.009	Polymorphism<br>0.993		
PREB<br>(2p23.3)	NM_001330486.1:c.1019C>T:<br>p.(Ser340Phe)	0.0000<br>(0.0001)	0.0000<br>(0.0002)	0.0019	0.0027	0.0046	Non-deleterious<br>9.72	–	–	–		
GTF3C2<br>(2p23.3)	NM_001521.3:c.323G>A:<br>p.(Arg108Gln)	0.0000<br>(0.00003231)	0.00005798	None	None	None	1% most deleterious<br>23.4	Possibly damaging<br>0.529	Tolerated<br>0.184	Polymorphism<br>0.884		
POTEE<br>(2q21.1)	NM_001083538.1:c.1995G>A:<br>p.(Met665Ile)	0.0031	0.0011	None	None	None	Non-deleterious<br>5.64	Benign<br>0.001	Damaging<br>0.000	Polymorphism<br>1.000		

TTN (2q31.2)	NM_133432.3:c.54400G>A: p.(Gly18134Arg)	None	0.0003	0.0009	0.0005	None	1% most deleterious 21.4	Possibly damaging 0.942	Damaging 0.009	Disease causing 1.000	Cardiomyopathy, familial hypertrophic, 9 (#613765)	AD
GPR55 (2q37.1)	NM_005683.3:c.260A>T: p.(Gln87Leu)	None	None	0.0009	0.0002	None	Non-deleterious 0.001	Benign 0.001	Tolerated 0.205	Polymorphism 1.000		
TMPRSS7 (3q13.2)	NM_001042575.2:c.1767A>G: p.(Ile589Met)	None	0.0000 (0.000004063)	0.0005	0.001	None	1% most deleterious 25.2	Probably damaging 0.994	Damaging 0.001	Disease causing 0.982		
SENP2 (3q27.2)	NM_021627.2:c.619G>A: p.(Gly207Ser)	None	0.0001	None	0.0002	0.0023	1% most deleterious 23.2	Probably damaging 1.000	Damaging 0.000	Disease causing 0.873		
TMEM175 (4p16.3)	NM_032326.3:c.1363G>A: p.(Val455Met)	0.0012	0.0009	None	None	None	Deleterious 11.8	Probably damaging 1.000	Damaging 0.011	Disease causing 0.925		
RGS12 (4p16.3)	NM_198229.2:c.4085C>G: p.(Pro1362Arg)	None	0.0000 (0.00001229)	None	None	0.0023	1% most deleterious 23.1	Probably damaging 0.971	Damaging 0.000	Disease causing 1.000		
ANKRD37 (4q35.1)	NM_181726.3:c.82G>A: p.(Asp28Asn)	None	0.0005	0.0018	0.0017	0.0023	1% most deleterious 25.7	Probably damaging 0.999	Damaging 0.000	−		
C5orf42 (5p13.2)	NM_023073.3:c.7478G>A: p.(Arg2493Gln)	0.0000 (0.00003229)	0.0004	0.0018	0.002	0.0023	Non-deleterious 9.04	Benign 0.001	Tolerated 0.439	Polymorphism 1.000		
PELO (5q11.2)	NM_015946.4:c.1120T>C: p.(Ser374Pro)	0.0000 (0.00003227)	0.0009	0.0009	0.0012	0.0023	1% most deleterious 23.4	Possibly damaging 0.909	Damaging 0.035	Disease causing 1.000		
SKIV2L2 (5q11.2)	NM_015360.4:c.43G>A: p.(Gly15Ser)	0.0012	0.0004	0.0009	0.0015	0.0023	Non-deleterious 9.23	Benign 0.001	Tolerated 0.415	Polymorphism 0.999		
COL4A3BP	NM_001130105.1:c.49_51del: p.(Pro17del)	None	0.0002	None	None	0.0023	−	−	−	−	Mental retardation, autosomal dominant 34 (#616351)	AD
MLIP (6p12.1)	NM_001281747.1:c.911A>G: p.(Gln304Arg)	None	0.0006	None	0.0027	0.0023	Non-deleterious 8.73	Benign 0.301	Tolerated 0.08	Disease causing 0.999		
FAM20C (7p22.3)	NM_020223.3:c.12G>A: p.(Met4Ile)	None	0.0000 (0.0000)	0.0014	0.0005	0.0023	Non-deleterious 0.087	Benign 0.000	Tolerated 1.000	Polymorphism 1.000	Raine syndrome (#259775)	AR
ELFN1 (7p22.3)	NM_001128636.2:c.1006C>T: p.(Pro336Ser)	None	0.0000 (0.0000)	None	None	0.0023	1% most deleterious 24.1	Probably damaging 1.000	Damaging 0.000	Disease causing 1.000		
ZNF716 (7p11.2)	NM_001159279.1:c.659A>G: p.(Lys220Arg)	None	None	None	0.0003	0.0023	Non-deleterious 9.61	Benign 0.027	Damaging 0.015	Polymorphism 1.000		
POM121 (7q11.23)	NM_001257190.2:c.2246T>C: p.(Met749Thr)	0.0006	0.001	None	None	0.0023	Non-deleterious 0.001	Benign 0.000	Tolerated 0.857	Polymorphism 1.000		
RAB11FIP1	NM_001002814.2:c.3274G>C: p.(Asp1092His)	0.0043	0.002	0.0036	0.002	0.0023	Deleterious 10.2	Benign 0.002	Tolerated 0.129	Polymorphism 1.000		
PXDNL (8q11.22)	NM_144651.4:c.2878C>G: p.(Arg960Gly)	0.0012	0.0007	0.0014	0.0005	0.0023	1% most deleterious 24.0	Probably damaging 1.000	Damaging 0.006	Disease causing 1.000		
PXDNL (8q11.22)	NM_144651.4:c.727G>A: p.(Val243Met)	None	None	None	None	0.0023	1% most deleterious 23.1	Probably damaging 0.952	Tolerated 0.074	Polymorphism 0.966		
CSMD3 (8q23.3)	NM_198123.1:c.746G>A: p.(Ser249Asn)	0.0025	0.0019	None	0.002	0.0023	1% most deleterious 24.5	Possibly damaging 0.753	Tolerated 0.103	Disease causing 0.926		
TYRP1 (9p23)	NM_000550.2:c.551T>C: p.(Ile184Thr)	None	0.0002	None	None	None	1% most deleterious 23.8	Probably damaging 0.975	Tolerated 0.104	Disease causing 1.000	Albinism, oculocutaneous, type III (#203290)	AR
ELP1 (9q31.3)	NM_003640.4:c.208C>T: p.(Arg70Cys)	0.0037	0.0031	0.0048	0.0029	0.0023	Deleterious 17.7	Benign 0.000	Tolerated 0.228	Polymorphism 0.999		
TXNDC8 (9q31.3)	NM_001003936.3:c.20A>G: p.(Asp7Gly)	None	0.0001	0.0005	0.0007	0.0023	Deleterious 11.4	Benign 0.002	Damaging 0.039	Polymorphism 1.000		

PSMD5 (9q33.2)	NM_005047.3:c.1199G>A: p.(Arg400His)	0.0000 (0.0000646)	0.0000 (0.00001661)	None	None	0.0023	1% most deleterious 28.5	Probably damaging 0.999	Tolerated 0.059	Disease causing 1.000		
MASTL (10p12.1)	NM_001172303.2:c.1544A>G: p.(Asp515Gly)	None	0.0006	0.0018	0.0027	0.0023	Non-deleterious 5.67	Benign 0.001	Tolerated 0.112	Polymorphism 1.000		
CPEB3 (10q23.32)	NM_014912.4:c.187C>G: p.(Pro63Ala)	0.0025	0.0019	0.0037	0.0035	0.0023	Non-deleterious 1.80	Benign 0.002	Tolerated 0.138	Polymorphism 0.977		
HPS1 (10q24.2)	NM_000195.4:c.1796C>T: p.(Thr599Met)	0.0000 (0.0004)	0.0001	None	0.0012	0.0023	1% most deleterious 30.0	Probably damaging 1.000	Damaging 0.001	Disease causing 1.000	Hermansky-Pudlak syndrome 1 (#203300)	AR
KRTAP5-6 (11p15.5)	NM_001012416.1:c.322A>C: p.(Ser108Arg)	None	None	None	0.0003	0.0023	Non-deleterious 1.29	Benign 0.343	–	Polymorphism 1.000		
OR52K2 (11p15.4)	NM_001005172.2:c.725C>T: p.(Thr242Ile)	0.0006	0.0001	0.0005	0.0005	0.0023	1% most deleterious 23.8	Benign 0.013	Damaging 0.005	Disease causing 0.999		
FAT3 (11q14.3)	NM_001008781.2:c.5344G>T: p.(Ala1782Ser)	None	0.0000 (0.000004098)	None	0.0002	0.0023	1% most deleterious 27.8	Probably damaging 1.000	Damaging 0.003	Disease causing 1.000		
CACNA1C (12p13.33)	NM_001167625.1:c.5564G>A: p.(Cys1855Tyr)	0.0037	0.0024	0.0005	0.0024	0.0023	Non-deleterious 5.86	Benign 0.000	Tolerated 1.000	Polymorphism 1.000	Brugada syndrome 3 (#611875)	–
PZP (12p13.31)	NM_002864.2:c.3800T>C: p.(Leu1267Pro)	None	0.0006	0.0039	0.0007	None	1% most deleterious 25.6	Probably damaging 1.000	Damaging 0.000	Disease causing 1.000		
PRB2 (12p13.2)	NM_006248.3:c.512G>A: p.(Arg171Gln)	0.0000 (0.0016)	0.0001	None	None	None	Non-deleterious 5.75	Benign 0.032	–	Polymorphism 1.000		
PPFIBP1 (12p11.22)	NM_003622.3:c.2245G>C: p.(Val749Leu)	0.0006	0.0028	0.0041	0.0015	0.0023	1% most deleterious 21.8	Benign 0.002	Damaging 0.006	Disease causing 1.000		
LGR5 (12q21.1)	NM_003667.3:c.417C>A: p.(Val749Leu)	None	0.0000 (0.00001222)	None	None	None	1% most deleterious 25.0	Probably damaging 0.990	Tolerated 0.192	Disease causing 0.885		
CIT (12q24.23)	NM_001206999.1:c.947T>C: p.(Met316Thr)	None	0.0004	0.0044	0.0039	0.0046	1% most deleterious 25.4	Benign 0.004	Damaging 0.002	Disease causing 1.000	Microcephaly 17, primary (#617090)	AR
TEP1 (14q11.2)	NM_007110.4:c.4370G>T: p.(Ser1457Ile)	None	0.0002	None	None	None	1% most deleterious 22.8	Possibly damaging 0.703	Damaging 0.011	Polymorphism 0.859		
DCAF4 (14q24.2)	NM_015604.3:c.935A>G: p.(Asn312Ser)	0.0000 (0.00003228)	0.0000 (0.00001625)	None	None	0.0023	Deleterious 14.8	Benign 0.043	Tolerated 0.190	Disease causing 0.656		
CDC42BPB	NM_006035.3:c.1552C>T: p.(Arg518Cys)	0.0000 (0.00006456)	0.0002	0.0018	0.0005	0.0023	1% most deleterious 26.7	Probably damaging 0.997	Damaging 0.011	Disease causing 1.000		
FAM189A1 (15q13.1)	NM_015307.1:c.1477G>T: p.(Val493Leu)	None	0.0009	0.0023	0.0022	0.0023	1% most deleterious 26.6	Probably damaging 0.999	Damaging 0.000	Disease causing 1.000		
STRA6 (15q24.1)	NM_001199040.1:c.25G>A: p.(Glu9Lys)	None	0.0004	0.0024	0.0007	0.0023	Deleterious 18.7	Benign 0.185	Damaging 0.000	Polymorphism 1.000	Microphthalmia, syndromic 9 (#601186)	AR
LINGO1 (15q24.3)	NM_032808.6:c.1274G>A: p.(Arg425His)	0.0000 (0.00006464)	0.0000 (0.00008808)	None	None	None	1% most deleterious 27.1	Probably damaging 0.997	Tolerated 0.059	Disease causing 1.000		
CHRNA3 (15q25.1)	NM_000745.3:c.1290G>A: p.(Arg425His)	None	0.0001	None	0.0007	None	Deleterious 15.9	Benign 0.158	Tolerated 1.000	Disease causing 1.000		
TICRR (15q26.1)	NM_001308025.1:c.1633C>T: p.(Arg545Cys)	0.0043	0.0019	0.0009	0.0007	None	1% most deleterious 21.5	Possibly damaging 0.563	Tolerated 0.067	Polymorphism 1.000		
FOXL1 (16q24.1)	NM_005250.2:c.458A>C: p.(Glu153Ala)	0.0031	0.0046	None	0.0002	None	1% most deleterious 20.8	Possibly damaging 0.816	Damaging 0.012	Polymorphism 1.000		
C3 (19p13.3)	NM_000064.3:c.1955A>C: p.(Gln652Pro)	0.0012	0.001	None	None	None	Non-deleterious 0.028	Benign 0.080	Tolerated 0.264	Polymorphism 1.000	C3 deficiency (#613779)	AR

ZNF709<br>(19p13.2)	NM_152601.3:c.635G>A:<br>p.(Arg212Gln)	0.0006	0.0000<br>(0.00001225)	None	None	None	1% most deleterious<br>17.5	Benign<br>0.169	Tolerated<br>1.000	Polymorphism<br>1.000		
PALM3<br>(19p13.12)	NM_001145028.1:c.425C>A:<br>p.(Ala142Asp)	None	0.0000<br>(0.00008399)	None	None	0.0046	1% most deleterious<br>23.3	Possibly damaging<br>0.835	Damaging<br>0.003	Polymorphism<br>1.000		
F2RL3<br>(19p13.11)	NM_003950.3:c.1115G>C:<br>p.(Gly372Ala)	None	0.001	None	None	0.0023	Non-deleterious<br>0.004	Benign<br>0.000	Tolerated<br>0.859	Polymorphism<br>1.000		
VASP<br>(19q13.32)	NM_003370.3:c.397C>T:<br>p.(Pro133Ser)	None	0.0000<br>(0.000005332)	None	None	0.0023	1% most deleterious<br>23.8	Probably damaging<br>1.000	Damaging<br>0.020	Disease causing<br>1.000		
POLD1<br>(19q13.33)	NM_001256849.1:c.511G>T:<br>p.(Ala171Ser)	None	0.0008	0.0009	0.0005	0.0023	1% most deleterious<br>20.3	Benign<br>0.051	Tolerated<br>0.416	Disease causing<br>0.996	Mandibular hypoplasia, deafness, progeroid<br>features, and lipodystrophy syndrome	AD
ZNF468<br>(19q13.41)	NM_001008801.1:c.1243G>A:<br>p.(Glu415Lys)	None	0.002	0.0024	0.0025	0.0046	1% most deleterious<br>13.1	Possibly damaging<br>0.886	–	Polymorphism<br>1.000		
RRBP1<br>(20p12.1)	NM_001042576.1:c.1951G>A:<br>p.(Glu651Lys)	None	0.0002	None	None	None	1% most deleterious<br>23.8	Benign<br>0.016	Tolerated<br>0.147	Polymorphism<br>1.000		
MYLK2<br>(20q11.21)	NM_033118.3:c.300G>T:<br>p.(Gln100His)	None	None	None	None	0.0023	Non-deleterious<br>13.0	Possibly damaging<br>0.61	Damaging<br>0.023	Polymorphism<br>1.000	Cardiomyopathy, hypertrophic, 1, digenic<br>(#192600)	AD
RALGAPB<br>(20q11.23)	NM_001282917.1:c.1448G>A:<br>p.(Arg483Gln)	None	0.0000<br>(0.00002844)	None	None	0.0023	1% most deleterious<br>22.5	Probably damaging<br>0.997	Tolerated<br>0.093	Disease causing<br>1.000		
MYT1<br>(20q13.33)	NM_004535.2:c.1709G>A:<br>p.(Arg570Lys)	0.0006	0.0008	None	0.0002	0.0023	1% most deleterious<br>28.1	Possibly damaging<br>0.877	Damaging<br>0.006	Disease causing<br>1.000		
CLDN17<br>(21q22.11)	NM_012131.2:c.71C>A:<br>p.(Thr24Lys)	0.0012	0.0023	0.0027	0.0012	0.0023	1% most deleterious<br>24.9	Probably damaging<br>0.976	Damaging<br>0.034	Polymorphism<br>1.000		
DGCR6L<br>(22q11.21)	NM_033257.3:c.544C>T:<br>p.(Leu182Phe)	None	0.0004	0.0036	0.0032	0.0046	1% most deleterious<br>22.5	Probably damaging<br>0.997	Tolerated<br>1.000	Disease causing<br>1.000		
FBLN1<br>(22q13.31)	NM_006487.2:c.1058G>A:<br>p.(Arg353His)	0.0000<br>(0.00009689)	0.0001	0.0014	0.0007	None	1% most deleterious<br>29.2	Probably damaging<br>0.999	Damaging<br>0.011	Disease causing<br>1.000	Synpolydactyly, 3/3'4, associated with<br>metacarpal and metatarsal synostoses	AD
TRMU<br>(22q13.31)	NM_001282784.1:c.628C>T:<br>p.(Pro210Ser)	None	0.0000<br>(0.000004066)	None	0.0012	None	1% most deleterious<br>16.1	Benign<br>0.001	Tolerated<br>0.260	Polymorphism<br>0.997	Liver failure, transient infantile (#613070)	AR

Shown are non-synonymous rare variants with minor allele frequencies (MAFs) <0.005 in gnomAD_genome_EAS, gnomAD_exome_EAS, HGVD, 2KJPN, and in-house 139 controls.
These variants have been selected as an autosomal-dominant or an X-linked recessive model. "None" indicates that the variant is not registered in the database.
The URLs utilized are as follows; *in silico* analyses were performed by using the default parameters.
1) gnom AD (Genome Aggregation Database): http://gnomad.broadinstitute.org/.
2) HGVD (Human Genetic Variation Database): http://www.hgvd.genome.med.kyoto-u.ac.jp/.
3) 2KJPN (Whole-genome sequences of 2,049 healthy Japanese individuals and construction of the highly accurate Japanese population reference panel): https://ijgvd.megabank.tohoku.ac.jp.
4) CADD (Combined Annotation–Dependent Depletion): http://cadd.gs.washington.edu/score (Current version: 1.3, GRCh37/hg19); PHRED scores of > 10–20 are regarded as deleterious, and those of > 20 indicates the 1% most deleterious.
5) Polyphen-2 Hum Var: http://genetics.bwh.harvard.edu/pph2/ (Current version: 2.2.2, GRCh37/hg19); HumVar scores were evaluated as 0.000 (most probably benign) to 1.000 (most probably damaging).
6) SIFT (Sorting Intolerant From Tolerant): http://sift.jcvi.org/ (Current version: Aug. 2011; GRCh37/Ensembl 63)); Scores of ≤0.05 and those > 0.05 are assessed as damaging and tolerated, respectively.
7) MutationTaster: http://www.mutationtaster.org/ (MutationTaster2, GRCh37/Ensembl 69); Alterations are classified as disease causing or polymorphisms,.
8) OMIM (Online Mendelian inheritance in man): http://omim.org/
9) UCSC genome browser: https://genome.ucsc.edu/.
* When the frequency in the east Asian (EAS) population (n = 811 for genomes and n = 8,624 for exomes) is 0.0000, the frequency in ALL populationsn (n = 15,496 for genomes and n = 123,136) for exomes is shown in parentheses.
